# Analysis of stability for nut yield and ancillary traits in cashew (*Anacardium occidentale* L.)

**DOI:** 10.1038/s41598-024-52030-6

**Published:** 2024-01-25

**Authors:** E. Eradasappa, G. S. Mohana, M. Poduval, K. Sethi, M. S. Aneesa Rani, I. K. Lourdusamy, S. Velmurugan, M. Manjusha, T. N. Raviprasad, C. Anilkumar

**Affiliations:** 1 ICAR-Directorate of Cashew Research, Puttur, India; 2 Regional Research Station, BCKV, Jhargram, India; 3 Cashew Research Station, OUAT, Bhubaneswar, India; 4 Regional Research Station, TNAU, Vridhachalam, India; 5 Regional Agricultural Research Station, KAU, Pilicode, India; 6 ICAR-National Rice Research Institute, Cuttack, India

**Keywords:** Genetics, Plant sciences

## Abstract

Cashew is cultivated in varied agro-ecological regions of India and yield levels vary with regions. Therefore, to identify stable genotype for yield, 18 genotypes were tested in four environments for nut yield and ancillary traits during 2008 to 2018 in randomized block design with two replications. The data of 6th annual harvest and cumulative nut yield of six years was analyzed employing additive main effect and multiplicative interaction (AMMI) and genotype and genotype by environment (GGE) methods. Analysis of variance for 6th annual harvest indicated significant differences (*p* < 0.01) for eight traits. Environments varied significantly (*p* < 0.01) for seven traits. Genotype by environment (G × E) interactions were significant (*p* < 0.01) for all traits. Analysis of variance for cumulative yield revealed significant variations between genotypes, environments, G x E interactions. Interaction principal component analysis (IPCA) 1 (84.39%) and IPCA 2 (10.27%) together captured 95% of variability. Genotypes, environments and G × E interaction were accounted for 16.18%, 4.50% and 77.22% respectively of total variation. The environment Pilicode discriminated better while Vridhachalam was representative. BPP-8 and Vengulra-7 were the winning genotypes in Bhubaneswar while Kanaka and Priyanka in Pilicode, Vengurla-4 in Jhargram and UN-50 in Vridhachalam. Therefore, promoting cultivation of these winning genotypes in the corresponding environments is highly recommended to enhance cashew nut production. As per ASV (AMMI stability value,) K-22-1 was stable genotype followed by Bhubaneswar-1. As per YSI (yield stability index), Bhubaneswar-1 was stable and high yielding followed by K-22-1 and BPP-8. Thus stable genotypes identified in this study viz., K-22-1 and Bhuvaneswar-1 are recommended for cultivation in west and east regions of India which have most cashew growing areas for increasing the cashew nut production.

## Introduction

Cashew (*Anacardium occidentale* L.) is cultivated in 46 countries of Africa, Asia, Latin America, and the Caribbean^1^. World over it is grown in an area of about 68.56 lakh hectares with a production of 38.52 lakh tonnes of raw nuts and average productivity of 561.9 kg/ha. Whereas in India, it is grown in an area of 11.84 lakh hectares with a production of 7.52 lakh tonnes of raw nuts with an average productivity of 635.1 kg/ha^2^. Cashew is a commercial plantation crop grown by marginal, small and big farmers as per their area availability for cultivation and most of the farmers sell the raw cashew nuts produced to processors and earn considerable profit. In India cashew has become a crop with high economic value and attained the status of an export–oriented commodity, earning considerable foreign exchange for the country. India exports cashew kernels to over 60 countries. Its major markets are US, Japan, Spain, France, Germany, UK as well as Middle East countries such as UAE and Saudi Arabia^3^. Cashew industry in India provides employment to more than 10 lakhs of people on farms and factories in rural areas^4^.

Cashew kernel contains proteins (21%), carbohydrates (22%), fat (47%), minerals and vitamins. Cashew kernel proteins contain all the essential amino acids and is comparable with other nuts like almond. Cashew kernel proteins are rich in acidic amino acids (38.78%). The major basic amino acids such as leucine and arginine are present to an extent of 22.23%. Cashew kernel does not contain any anti-nutritional factors. Cashew kernel contains sizeable quantity of vitamin E, a naturally occurring antioxidant (210 mg/IOO g) and few water soluble B vitamins such as thiamine, riboflavin, niacin, biotin, folic acid, vitamin B6, B12 and pantothenic acid. Cashew kernel is rich in potassium and phosphrous. As cashew kernel oil contains vitamin E, it has use in cosmetic industry. Cashew shell contains cashew nut shell liquid (CNSL) to an extent of 35% by weight. Commercially CNSL is extracted by expeller and the residue after extraction of CNSL is used as fuel for generation of steam required for steam boiling of the cashew nut. CNSL contains 90% of anacardic acid, 5% each of cardanol and cardol. Anacardic acid finds extensive industrial application in textile, timber protection, preparation of formaldehyde resins, abrasives, brick lining, and ship building because of its high antimicrobial properties. The pseudo fruit which is called as cashew apple is a juicy fibrous nutritious fruit. It contains sugars, amino acids, tannin, ascorbic acid (Vitamin C) and crude fibre. It is rich in ascorbic acid (240 mg/IOO g) which is almost six times that of citrus fruits (40 mg/100 g). Its juice can be used for preparation of RTS, Jam, Jelly, Syrups and alcoholic beverages^5^.

Cashew is cultivated in different agro-ecological regions of India and farmers in one region get more yield while in other regions less yield for the same variety. This is attributed to the phenomenon of genotype by environment interaction (G × E) effect that is the influence of environmental conditions on the phenotypic expression of the genotypes. Previous studies on cashew performance in diverse environments indicated significant G × E interaction effect for nut yield and its related traits^6–10^. Similar studies were carried out in mango (*Mangifera indica* L.) which belongs to same cashew family Anacardiaceae^11–13^. The G × E interaction effect arises due to variations in the sensitivity of genotypes to the conditions in the environments they are grown^14^ and it affects the efficiency of selection of superior genotypes^15^.

Most of the crop breeding programmes aim at assessing the yield performance of genotypes across several environments to identify genotype that is high yielding and adaptive to one specific environment or more than one environment (stable). This is an important approach for improving cashew nut production in India as its domestic production is inadequate to meet the processing demand. The stable performance of genotypes in different environments decides the efficiency and success of selection^16^ and hence genotypes are evaluated in different environments to find out their adaptability and stability^17^. Many biometrical models are developed to estimate the G × E effect and stability of genotypes. Among them, additive main effect and multiplicative interaction (AMMI) analysis^18^ is a versatile statistical model as it has the capacity to capture good amount of G × E effect and enables the crop breeders to interpret the data proficiently and choose the variety suitable for each environment. Besides, GGE (genotype and genotype by environment) bi-plot analysis^19^ is being used to supplement the AMMI analysis which helps in removing the effect of environment and integrates genotypic effect with G × E interaction effect^6^. The AMMI model estimates the stability of the genotypes based on AMMI stability value (ASV)^20^ which is calculated using IPCA1 and IPCA2 (interaction principal components axes 1 and 2, resp.) scores for each genotype^21^. The least ASV score of genotypes indicates genotypes are widely adapted. It is said that stability per se may not give considerable information regarding the level of yield^21,22^ and thus they proposed yield stability index (YSI) that is calculated as sum of the rankings due to ASV scores and yield. The genotypes with lower YSI values denote high yielding and stable^22–26^. Hitherto, AMMI models were applied in cashew breeding programme in Nigeria^6^ and Benin^9^ and we are applying this model in Indian cashew breeding for the first time. With this backdrop, 18 cashew genotypes developed from different cashew research centers were evaluated in four environments in India for nut yield and related traits with the aim of understanding the influence of G × E effect and to identify stable and high yielding genotype for nut yield.

## Materials and methods

### Study environments

**Figure 1:**
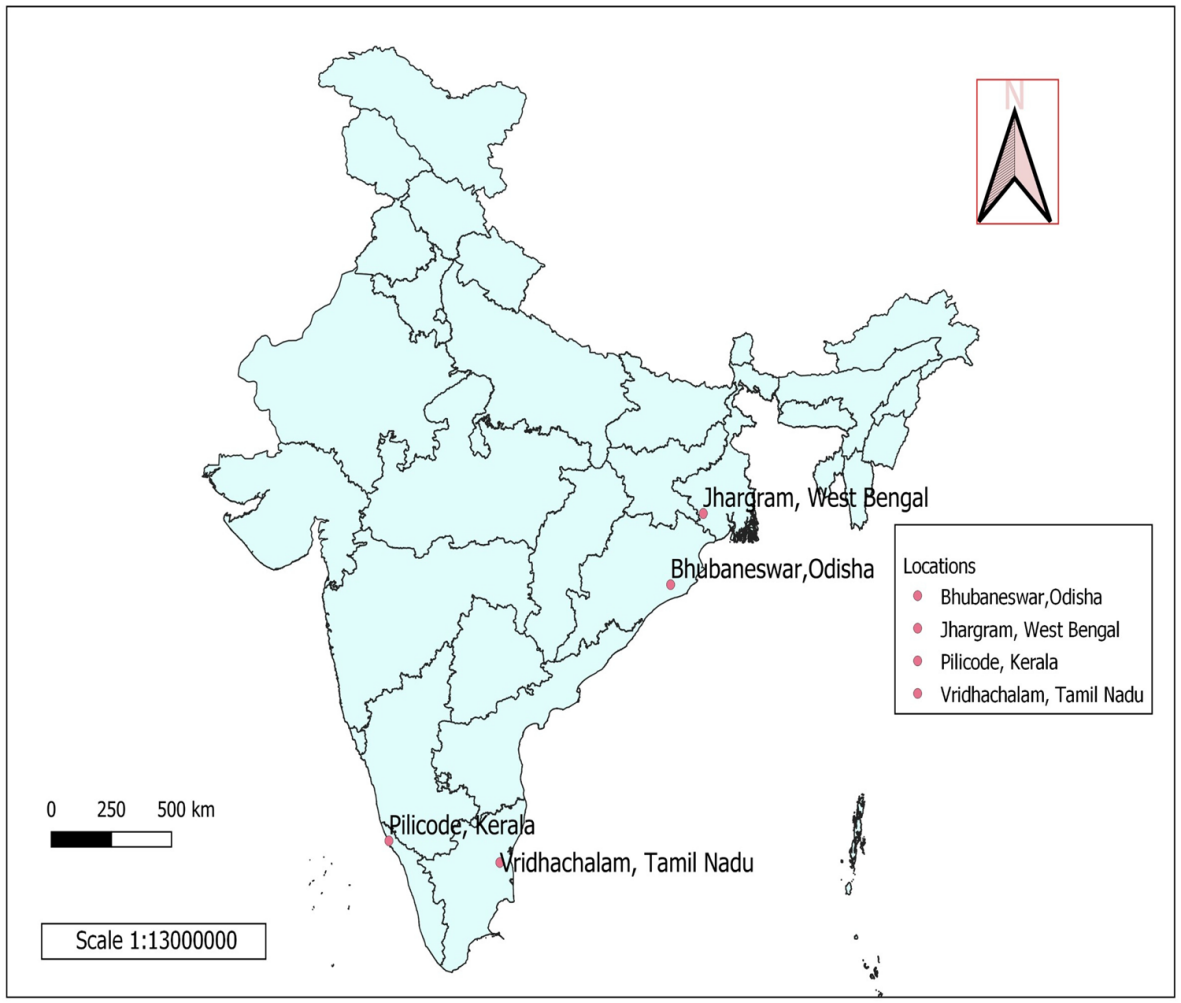
India map showing the 4 experimental locations (environments) of the study.

The trial was conducted in four environments (Fig. 1) which are the cashew research centers under All India Coordinated Research Project (AICRP) on Cashew in India viz., (1) Regional Agricultural Research Station, Pilicode (KAU), (2) Regional Research Station, Vridhachalam (TNAU), (3) Bhubaneswar (OUAT) and (4) Regional Research Station, Jhargram (BCKV). The details of climate and soil type is presented in the Table 1.

**Table 1:** Climate and soil type of study environments.

Environment	Lat-longitude	Altitude from MSL	Rainy season	Average annual rainfall (mm)	Mean min temp	Mean max temp	Soil type
Pilicode	13°N- 75°E	15 m	June to Oct	3379	23 °C	33 °C	laterite
Vridhachalam	11.30°N -79.26°E	42.67 m	June-Sep & Oct-Dec	1020	19 °C	42 °C	laterite and sandy
Jhargram	22° 30’N -87°E	81 m	June to October	1400	4 °C	46 °C	sandy loam
Bhubaneswar	20°15’ N- 82°52’ E	25.5 m	June to Oct	1640	22 °C	32 °C	sandy loam

### Experimental material

Eighteen cashew varieties developed different cashew research centers viz., Amrutha, Bhaskara, Bhubaneswar-1, BPP-8, Dhana, Goa-1, K-22-1, Kanaka, Madakkathara-1, Madakkathara-2, NRCC Sel-2, Priyanka, Ullal-3, Ullal-4, UN-50, Vengurla-4, Vengurla-7, VRI-3 formed the experimental material for the study. The list of genotypes and their source is presented in the Table 2^27^. ICAR- Directorate of Cashew Research (DCR), Puttur, Karnataka is the nodal institute for the project AICRP on Cashew in India and the research stations of State Agricultural Universities (SAUs) are the coordinating centers. Permission was obtained from the different centers of SAUs for collection of varieties for conducting the Multi-Location Trial. The experimental research and field studies including collection of plant material in the present study comply with relevant institutional, national, and international guidelines and legislation.

**Table 2:** List of cashew genotypes evaluated in MLT-V and their source.

S. No	Genotype	Source
1	BPP-8	Cashew Research Station, Bapatla
2	Bhubaneswar-1	Cashew Research Station, OUAT, Bhubaneswar
3	Madakkathara-1	Cashew Research Station, Madakkathara, KAU
4	Madakkathara-2	Cashew Research Station, Madakkathara, KAU
5	K-22-1	Cashew Research Station, Madakkathara, KAU
6	Dhana	Cashew Research Station, Madakkathara, KAU
7	Kanaka	Cashew Research Station, Madakkathara, KAU
8	Priyanka	Cashew Research Station, Madakkathara, KAU
9	Amrutha	Cashew Research Station, Madakkathara, KAU
10	Vengurla-4	Regional Fruit Research Station, Vengurla, KKV
11	Vengurla-7	Regional Fruit Research Station, Vengurla, KKV
12	VRI-3	Regional Research Station, Vridhachalam,TNAU
13	NRCC Sel-2	ICAR-Directorate of Cashew Research, Puttur, Karnataka
14	Ullal-3	Agricultural Research Station, Ullal, Karnataka
15	Ullal-4	Agricultural Research Station, Ullal, Karnataka
16	UN-50	Agricultural Research Station, Ullal, Karnataka
17	Goa-1	ICAR-CCARI, Goa
18	Bhaskara	ICAR-Directorate of Cashew Research, Puttur, Karnataka

### Experimental design and layout

The experiment was designated as Multi-Location Trial-V (MLT-V) and started in the year 2008 in three locations Bhubaneswar, Pilicode, Vridhachalam and in the year 2010 at Jhargram location. The genotypes were planted at 7 m × 7 m spacing in randomized block design (RBD) with two replications. There were three trees / genotype in each replication. Recommended horticultural practices and plant protection measures were adopted for raising the plants and protecting the plantation.

### Data collection

The data of nut yield and other traits was collected from 3^rd^ year after planting as per the methodology described in the experimental manual on cashew by the National Research Center for Cashew^28^. The height of tree was measured using marked bamboo pole / PVC pipe from the ground to the tip of the main branch in meters. The circumference of the trunk or main stem at the collar region (15 cm above ground level) was measured using measuring tape in centimeters. The canopy spread in east–west direction and north–south direction was measured using measuring tape and average of the two directions was expressed as tree spread in metre. The number of flowering laterals were counted from one square metre area of canopy in four directions using frame of one square metre bamboo poles and the mean of four directions was arrived. For sex ratio, three panicles were selected from the observational tree and they were tagged. The numbers of bisexual and male flowers appearing in each panicle were counted on alternate days and the counted flowers were removed. This was done till the end of flowering in those panicles. The total number of bisexual and male flowers in each panicle was obtained. The mean of bisexual flowers per panicle and mean of male flower per panicle was determined. The sex ratio was calculated as

$$\mathrm{Sex\, Ratio}= \frac{\mathrm{mean\, of\, bisexual\, flowres}}{\mathrm{mean\, of\, male\, flowers}}$$

In case of nuts per panicle, matured nuts per panicle were counted in 20 panicles per tree in four directions and mean was taken. For collecting data of nut weight, 50 raw nuts were sun dried for three days and weight was taken in grams. The mean weight of nut was calculated as

$$\mathrm{Nut\, weight}= \frac{\mathrm{weight\, of\, }50\mathrm{ nuts}}{\mathrm{number\, of\, nuts}}$$

For shelling percentage, 50 raw nuts were weighed and weight was taken in grams. These nuts were shelled using shelling machine. Weight of kernel with testa and weight of shells obtained after shelling were recorded separately. The shelling percentage was calculated as

$$\mathrm{Shelling\, percentage}= \frac{\mathrm{weight\, of\, kernels\, with\, testa}}{\mathrm{weight\, of\, nut\,}}\mathrm{ x\, }100$$

For annual nut yield, matured nuts were harvested separately from three trees per genotype in each replication during the entire period of fruiting season from February-May every year. The harvested nuts from each tree were sun dried for three days and weighed for each genotype separately and expressed as nut yield / tree in kg. Likewise nut yield was collected for six years in each environment. The nut yield of six years was added to obtain cumulative nut yield / tree in kg.

### Statistical analysis

The data of nine traits collected during 6th annual harvest (that is in eight-year-old trees) and the data of cumulative nut yield of six years from the four environments was subjected for AMMI and GGE bi-plot analyses using the software GENSTAT-18.1 version by VSN International Ltd. (www.vsni.co.uk/software/genstat). As the performance of genotypes at one location was known, the genotypes were treated as fixed variables and test environments were considered as random variables while analyzing the data. The additive main effects of genotypes and environments were fitted by univariate ANOVA and G × E interaction was fitted by principal component analysis based on the AMMI II model. The graphical representations of GGE biplots were drawn using GEA-R software^29^. Stability of genotypes was assessed using the following two methods.

### AMMI stability value (ASV)

$${\text{ASV}}= \sqrt{[ \{{\text{IPCA}}1\mathrm{ SS }/\mathrm{ IPCA}2\mathrm{ SS}\} \times ({\text{IPCA}}1{\text{score}})]^2 + ({\text{IPCA}}2{\text{score}})^2}$$

where, IPCA1 SS and IPCA2 SS are sum of squares corresponding to first two IPCA’s. Smaller ASV values denote the greater stability of genotypes across environments^21^.

**Yield stability index (YSI):** This method enables simultaneous selection of genotype for high yield as well as stability, as it takes into account both yield and stability in a single measure^22^. YSI was estimated using the following formula.

$${\text{YSI}} = {\text{ RASV}} + {\text{RY}}$$

where, RASV is the rank of the genotypes based on the ASV and RY is the rank of the genotypes based on yield across environments.

## Results

### Analysis of traits of 6th annual harvest

#### Combined analysis

Combined AMMI analysis of variance over four environments for nine traits is presented in Table 3. Genotypes showed significant differences (*P* ≤ 0.01) for eight traits but not for flowering laterals / m^2^. Environments presented significant differences (*P* ≤ 0.01 or *P* ≤ 0.05) for seven traits but not for nut weight and shelling percentage. G x E interactions were significant (*P* ≤ 0.01) for all the traits. The variation contributed by the effect of genotype varied from 6.39% (flowering laterals / m^2^) to 37.56% (nut weight) while the variation due to the effect of the environment was between 4.3% (shelling percentage) and 44.99% (tree height). The sums of variations of IPCA1 and IPCA2 ranged from 83.66% (shelling percentage) to 96.91% (sex ratio).

**Table 3:** Combined AMMI analysis of variance over four environments for traits.

Source	DF		Flowering Laterals/m^2^		% Variation		Stem Girth (cm)		% Variation	Tree Height (m)		% Variation		Nut Weight (g)		% Variation		Nuts/panicle		% Variation
Genotypes (G)	17		19.6 ns		6.39		175.8**		15.82	0.833**		8.37		5.691**		37.56		10.14**		16.29
Environments (E)	3		627.2**		36.12		1693.9**		26.90	25.374**		44.99		15.287 ns		17.81		53.95*		15.30
Blocks	5		135.1**		10.38		368**		9.97	5.311**		12.55		12.043**		18.70		20.57**		7.78
G x E	51		30.4**		29.77		121.0**		32.66	0.691**		20.83		1.216**		24.08		10.70**		51.58
IPCA1	19		48.1**		58.99		210.0**		64.67	1.350**		72.77		1.926**		59.00		19.45**		67.75
IPCA2	17		28.1**		30.75		73.8**		20.32	0.380**		18.33		0.921**		25.25		6.49**		20.22
Residual	15		10.6		10.25		61.7		14.99	0.210		8.94		0.651		15.75		4.38		12.03
Error	68		13.3		17.35		41.3		14.86	0.330		13.24		0.070		1.84		1.41		9.04

Source		DF		Shelling Percentage		% Variation		Sex Ratio			% Variation		Canopy Spread (m)		% Variation		Nut Yield (kg/tree)		% Variation	
Genotypes		17		7.074**		18.44		0.03547**			15.94		1.466**		15.99		12.12**		13.51	
Environments		3		9.412 ns		4.32		0.36939**			29.28		12.771**		24.58		117.54**		23.11	
Blocks		5		4.690**		2.88		0.01384**			1.45		1.408**		3.61		0.84 ns		0.22	
G x E		51		8.349**		65.28		0.03627**			48.89		1.125**		36.81		14.76**		49.35	
IPCA1		19		12.326**		55.00		0.06615**			67.94		1.928**		63.87		23.67**		59.74	
IPCA2		17		7.227**		28.86		0.03153**			28.97		0.916**		27.14		12.33**		27.84	
Residual		15		4.583		16.13		0.00378			3.08		0.344		8.99		6.23		12.42	
Error		68		0.872		0.46		0.00247			4.40		0.435		19.00		3.10		13.81	

DF: degreed of freedom, G × E: genotype by environment interaction, IPCA: interaction principal component analysis, * significant at *P* ≤ 0.05 **: significant at *P* ≤ 0.01, ns: non-significant.

### Ranking of genotypes vis-a vis environment for traits

The ranking of genotypes in each environment for the data of nut yield and other traits taken during 6th annual harvest is presented in Table 4 (supplementary file). It showed that BPP-8 ranked first for nut yield (16.34 kg /tree), VRI-3 for number of flowering laterals per sqm (23.29), Kanaka for sex ratio (0.90) and Vengurla-7 for stem girth (76.25 cm) in the environment Bhubaneswar. VRI-3 ranked first for plant height (3.01 m) and Madakkathara-1 for tree spread (4.30 m) in the environment Vridhachalam. Priyanka ranked foremost for nut weight (10.88 g) while Madakkathara-1 for nuts per panicle (13.04) in the environment Pilicode. VRI-3 ranked top for shelling percentage with a mean of 35.83% in the environment Jhargram.

### Analysis of cumulative nut yield

#### Combined AMMI ANOVA

The combined AMMI analysis of variance over four environments for cumulative nut yield per tree of six years is presented in Table 4. The mean squares due to genotypes, environments, genotype and environment interactions (G × E) were all significant (*P* ≤ 0.01). The variations explained by genotypes, environments and G × E were 16.18%, 4.5%, 77.22% respectively, out of the total variation (Table 5). The sum of variations of IPCA1 and IPCA2 is about 94.66% in the G × E interaction component.

**Table 4:** Combined AMMI analysis of variance for cumulative nut yield of six years.

Source of variation	DF	SS	MS	F Value	F pr
Genotypes (G)	17	1662	97.70**	36.59	< 0.001
Environments (E)	3	462	153.8**	17.67	< 0.001
Blocks	5	35	8.7**	3.26	0.0166
G x E	51	7932	155.5**	58.22	< 0.001
IPCA1	19	6694	352.3**	131.88	< 0.001
IPCA2	17	815	47.9**	17.94	< 0.001
Residual	15	423	28.2	10.57	< 0.001
Error	68	182	2.7		
Total	143	10,272	71.8		
	R^2^ = 0.99	CV(%) = 7.39	Grand mean = 18.01		

DF: degreed of freedom, SS: sum of squares, MS: mean squares, G × E: genotype by environment interaction, IPCA: interaction principal component analysis, **: significant at *P* ≤ 0.01, CV = coefficient of variation, R^2^ = coefficient of determination.

**Table 5:** Variation Explained (%) by sources of variation for cumulative nut yield.

Source of variation	SS	Variation Explained (%)
Genotypes (G)	1662	16.18
Environments (E)	462	4.50
Blocks	35	0.34
G x E	7932	77.22
IPCA1	6694	84.39
IPCA2	815	10.27
Residual	423	5.33
Error	182	1.77
Total	10,272	100.00

DF: degreed of freedom, SS: sum of squares, G × E: genotype by environment interaction, IPCA: interaction principal component analysis.

### GGE bi-plot analyses

#### Discriminativeness vs. representativeness

**Figure 2:**
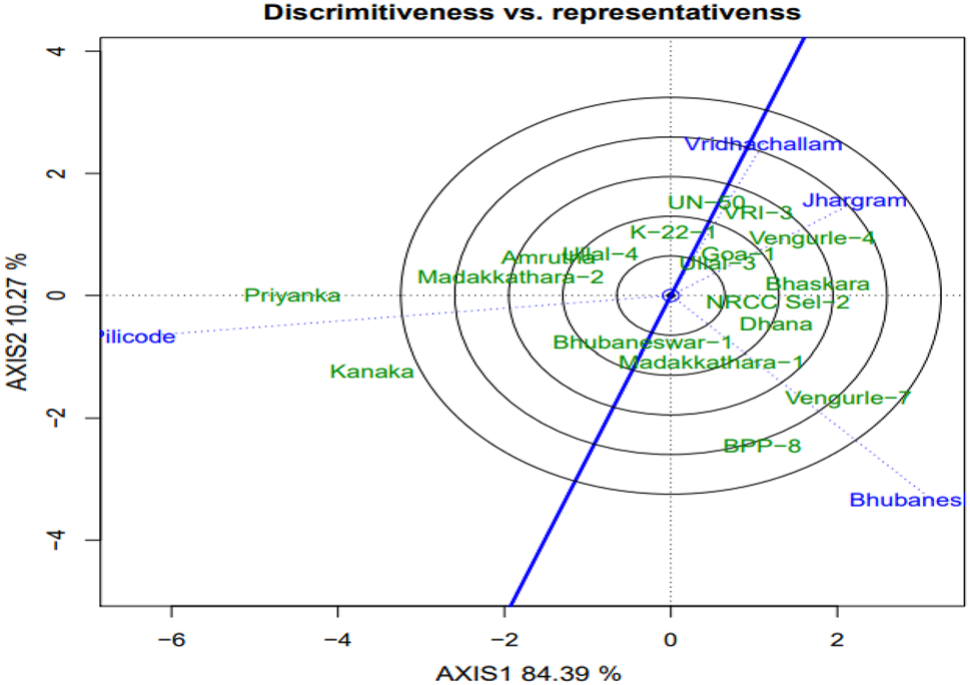
GGE bi-plot of cashew genotypes displaying discriminativeness vs. representativeness.

The ability of environment to differentiate genotype is called discriminativeness while the ability of environment to represent all the environments evaluated is known as representativeness. These two attributes are illustrated in the Fig. 2. Lines that connect between origin and environment are the environment vectors. Environments vectors with shorter length denote less discriminating ability while those with longer vector length show higher discriminating ability. In this study, the environment Pilicode showed higher discrimination of genotypes compared to other environments. The line with single arrow passing through the origin and the small concentric circle indicates average value of the environment and is referred to as average environment coordination (AEC) abscissa or average environment axis (AEA). An environment that has a small angle between its vector and the AEA is more representative^30^. In our study, the environment Vridhachalam is found to be more representative as it formed smaller (acute) angle with AEA. Acute angle between two environment vectors denote positive association or similarity while obtuse angle indicate negative association and right angle depict no association. The angles between the vectors of Vridachallam and Jhargram, Jhargram and Bhubaneshwar, Bhubaneswar and Pilicode were acute suggesting their effects were similar for genotype expression. The angle between Pilicode and other three environments was obtuse displaying negative correlation or dissimilarity.

#### Comparing genotypes with ideal genotype

**Figure 3:**
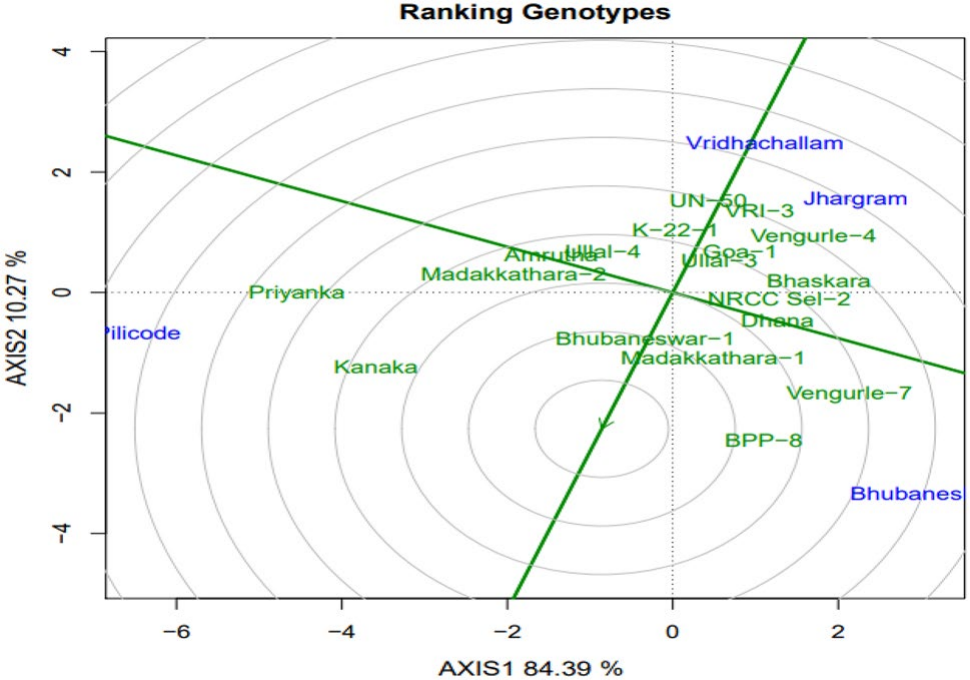
GGE bi-plot of cashew genotypes displaying ranking of genotypes in relation to ideal genotype.

Ideal genotype is the genotype that has both greater mean performance as well as greater stability for a specific trait evaluated over mega- environment^30^. It is represented by the point on the AEC abscissa at the center of concentric circles in the GGE bi-plot. The genotypes that are located close to the ideal genotype are considered as the best. In the present study, genotypes Madakkathara-1, BPP-8 and Bhubaneswar-1 were considered as best for cumulative nut yield / tree (Fig. 3).

#### Means vs. stability

**Figure 4:**
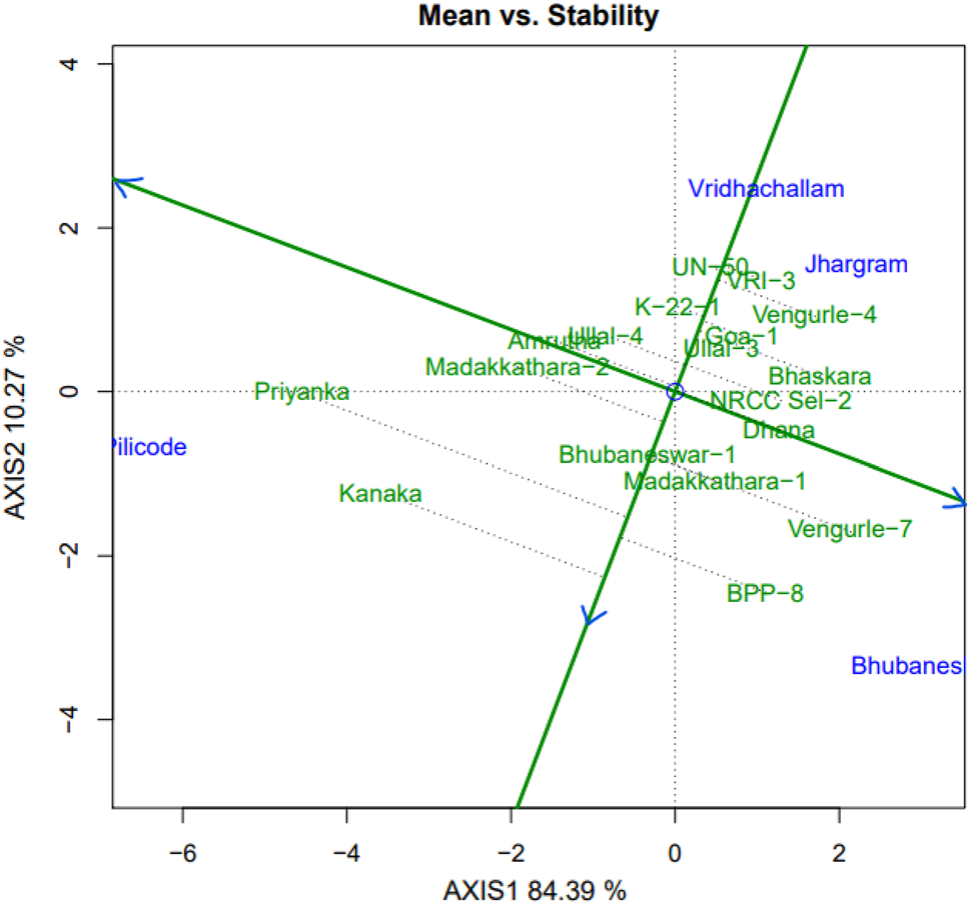
GGE bi-plot of cashew genotypes showing mean vs. stability for cumulative nut yield per tree.

The GGE bi-plot of means versus stability (Fig. 4) consists of two lines viz., i) the average environment axis (AEA) or average environment coordination (AEC) abscissa and ii) the AEC ordinate. AEA is the line with a single arrow that passes through the origin of bi-plot and the theoretical average of the environment denoted by small circle. The direction of the arrow on the AEA indicates greater mean performance for the trait or main effect of genotype^31^. Genotypes situated towards the direction of arrow indicate greater mean values while those located in the opposite direction of arrow denote lower mean values. Thus in the present case variety Kanaka has greater mean value while UN-50 has lowest mean value. The AEC ordinate also passes through the origin and is perpendicular to the AEA. The two ends of this ordinate suggest poor stability or greater G × E effect in either direction. The relative lengths of projections of genotypes from the AEA measure their stability. Shorter the projection of genotype greater is the stability and vice-versa^32^. In the present study, genotypes Bhubaneswar-1 and K22-1 presented shorter projections suggesting greater stability for nut yield /tree. Whereas the genotype Priyanka had more projection length indicating poor stability.

### Which–won–where

**Figure 5:**
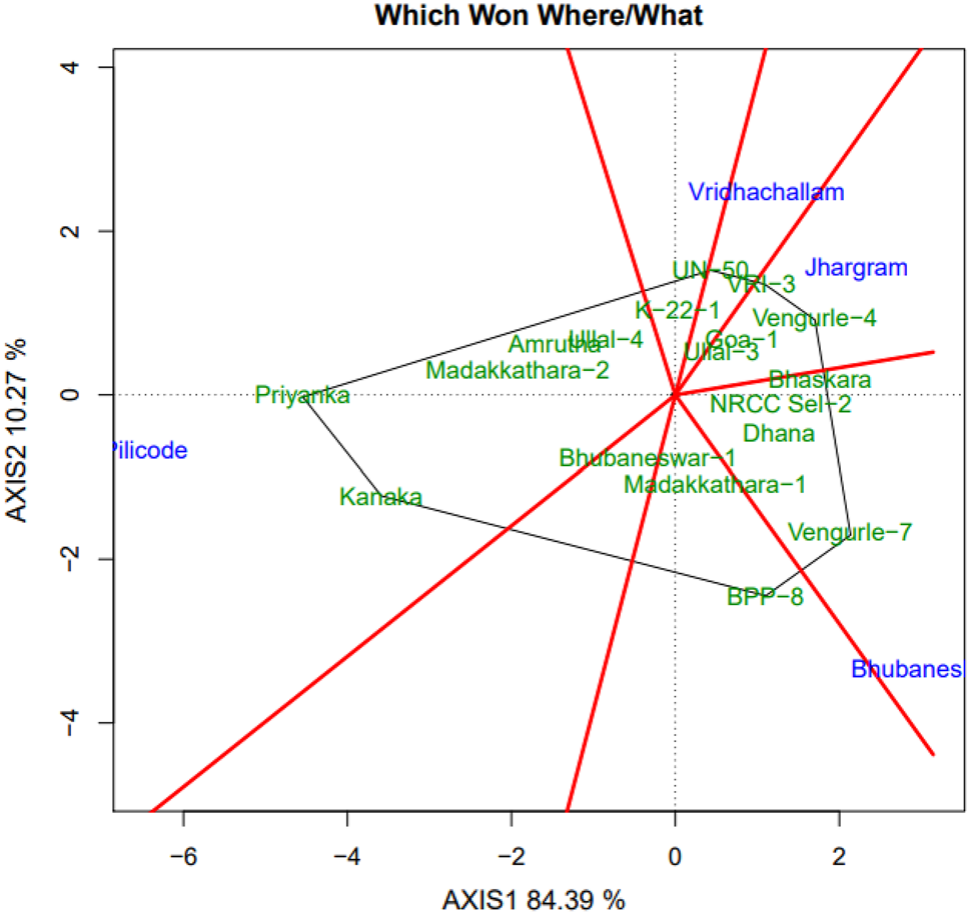
GGE bi-plot of cashew genotypes showing ‘which-won-where’ for cumulative nut yield per tree.

Which –won –where pattern of genotype is given by the polygon view of the bi-plot and it describes the performance of genotypes according to environments (Fig. 5). Genotypes which perform well in a particular environment are called winning genotypes. The polygon is divided into different sectors by the lines drawn from the origin of the bi-plot and perpendicular lines drawn from the origin to the sides of the polygon. The genotypes that are situated on the vertices of polygon are the winning genotypes^33^. In the present study, BPP-8 and Vengulra-7 were the winning genotypes in Bhubaneswar while Kanaka and Priyanka were the winning genotypes in Pilicode, Vengurla-4 in Jhargram and UN-50 in Vridhachalam.

#### Ranking of genotypes as per ASV and YSI

The genotypes were ranked for their stable performance across the environments using ASV and YSI separately (Table 6). Low ASV scores denote the most stable genotypes and hence ranks were assigned accordingly. K-22-1 ranked first according to ASV followed by Bhubaneswar-1. According to YSI, genotypes with lesser YSI values are stable for yield. On the basis of YSI values, Bhubaneswar-1 ranked first and the second rank was shared by K-22-1 and BPP-8. It is interesting to note here that K-22-1 ranked first as per ASV and ranked second as per YSI.

**Table 6:** Ranking of genotypes as per ASV and YSI for cumulative nut yield.

S. No	Genotype	Mean	Rank (A)	ASV Score	ASV Rank (B)	YSI (A + B)	YSI Rank
1	BPP-8	23.26	3	9.38	9	12	2
2	Bhubaneswar-1	20.13	5	2.81	2	7	1
3	Madakkathara-1	14.78	16	4.19	4	20	6
4	Madakkathara-2	17.58	10	15.79	15	25	9
5	K-22–1	15.99	11	1.08	1	12	2
6	Dhana	15.65	13	10.42	10	23	9
7	Kanaka	24.70	1	29.48	17	18	5
8	Priyanka	24.00	2	37.32	18	20	6
9	Amrutha	17.65	9	12.07	12	21	7
10	Vengurla-4	17.85	8	14.02	13	21	7
11	Vengurla-7	21.27	4	17.64	16	20	6
12	VRI-3	15.59	14	9.06	8	22	8
13	NRCC Sel-2	15.01	15	10.58	11	26	11
14	Ullal-3	15.75	12	4.64	5	17	4
15	Ullal-4	18.60	7	6.97	7	14	3
16	UN-50	12.74	18	3.85	3	21	7
17	Goa-1	14.61	17	6.74	6	23	9
18	Bhaskara	20.08	6	14.51	14	20	6

## Discussion

### Traits of 6th annual harvest

Genotypes varied for eight traits but not for flowering laterals / m^2^. Nut weight and shelling percentage were not varied in the environments indicating their qualitative nature. All the nine traits were significantly influenced by G × E justifying the need for the assessment of stability. The ranking of genotypes in each environment for the traits indicated that their expression varied with environments and prevailing conditions in that environment. For instance, the maximum number of flowering laterals per m^2^ in the variety Vridhachalam-3 and the maximum sex ratio in Kanaka in the environment Bhubaneswar could be due to the conducive mean maximum (32 °C) and minimum temperatures (22 °C) prevailing in that location. Similarly, the maximum stem girth and tree spread in Vengurla-7 in Bhubaneswar could be attributed to good soil fertility. The maximum tree height in Madakkathara-2 and nuts per panicle in Madakkathara-1 and nut weight in Priyanka in Pilicode could be due to very high average annual rainfall (3379 mm) and ideal mean maximum (33 °C) and minimum temperature (23 °C) prevalent in that location. The maximum shelling percentage in VRI-3 in Jhargram might be due to better drying of nuts due to high temperatures (46° C) during post- harvest period in the hot and dry months of May and June. In light of the variable response of genotypes for different traits, it is worth to consider the suggestion given for analysis of environmental factors such as rainfall, sunlight, temperature and water holding capacity of soil during various phenological stages to understand the causative factors of G × E interaction^34,35^. Many studies have showed the influence of environment on the growth, development and production of cashew trees^6,8,9,36–39^.

The varieties that showed very good performance for ancillary traits in the study like Vridhachalam-3 (number of flowering laterals per m^2^, shelling percentage), Kanaka (sex ratio), Vengurla-7 (stem girth), Madakkathara-2 (tree height), Madakkathara-1 (nuts per panicle) and Priyanka (nut weight) can be utilized as donor parents for improvement of those traits in the cashew breeding programme as they are positively correlated with nut yield.

### Cumulative nut yield of six years

The highly significant G × E interaction for cumulative nut yield indicates the differences in adaptation by the genotypes or the effect of environment on the performance of genotypes and justify the necessity to identify environment specific genotypes. The variability captured by IPCA 1 and IPCA 2 was about 95% in the G × E interaction for nut yield and it demonstrated the adequacy of the AMMI II model. These results are corroborated with report of 99.1% for the first two IPCAs in cashew in Nigeria^1^. The very high (77.22%) extent of variation explained by G × E interaction underscored the importance of the study. The variation explained by genotype (16.18%) and environment (4.5%) was not so high. In Nigerian cashew breeding programme^1^, reported variation explained by genotype of 53.53%, environment of 4.35% and G × E interaction of 24.69%. In mango which belongs to cashew family Anacardiaceae, the variation explained by genotypes, environments and G × E interaction were 6.95%, 37.78%, and 42.81% ^40^.

### Stability

In cashew, the cumulative nut yield of six years i.e., from 3rd year (starting year of fruiting) to 8th year of planting is considered for evaluating the yield performance of genotypes. Thus, stability of genotypes was assessed for cumulative nut yield. In light of the better discrimination by the environment Pilicode for cumulative yield, it becomes the choice of environments for testing the genotypes. The variety K-22-1 was the most stable genotype followed by Bhubaneswar-1 as per ASV. It is said that stability only for yield performance does not merit selection as a constantly low yielding genotype can still be stable^30^. Besides, it is said that the most stable genotype based on ASV does not show the best yield performance in some cases^41^. It is true in the present study as the yield of K-22-1 is much lower than Bhubaneswar-1, yet it is found stable genotype based on ASV. Thus, it is imperative to ponder index YSI. Consequently, YSI was estimated for cashew genotypes and it uncovered Bhubaneswar-1 as the most stable and high yielding genotype followed by BPP-8 and K-22-1, both of which shared second rank.

Identification of stable genotype based on multiple criteria is ideal to choose a stable genotype for cultivation as it accounts for per se performance along with its stability. The genotypes were compared for stability using AMMI stability value and GGE biplots, and for yield using yield stability index. Genotypes K-22-1 and Bhabaneswar-1 showed higher rank for ASV value and YSI values. Further, these genotypes were near to origin in the GGE biplots where Bhubaneswar-1 was close to ideal genotype. Apart from that, these genotypes showed higher mean performance as indicated in the mean vs stability plot. On the other hand, genotype BPP-8 showed higher YSI rank and found ideal for Bhubaneswar environment as it was very close to Bhubaneswar in which-won-where plot. Therefore, the genotypes K-22-1 and Bhubaneswar-1 were found highly stable for cultivation across environments and BBP-8 was ideal for Bhubaneswar environment.

## Conclusion

The findings of this study in cashew revealed the significant genotype by environment interaction for most of the traits, annual and cumulative nut yields implying the need for testing the genotypes bred in multi-environments. The study identified genotypes Bhubaneswar-1, BPP-8 and K-22-1 as stable and high yielding for cumulative nut yield. Thus these stable genotypes identified are recommended for cultivation in both west and east coast regions of India which cover more cashew area to increase the production of raw cashew nuts.

### Supplementary Information

Supplementary Information.

## Data Availability

The datasets generated during and/or analyzed during the current study are available from the corresponding author on reasonable request.
