# Supplementary material for: Analysis of stability for nut yield and ancillary traits in cashew (Anacardium occidentale L.)
Source: Sci Rep. 2024 Jan 25;14:2127. doi: 10.1038/s41598-024-52030-6 (PMC10808333; doi:10.1038/s41598-024-52030-6)
Supplement: Supplementary file 1 — Supplementary Information. [file 41598_2024_52030_MOESM1_ESM.docx]

**Table 4: (Supplementary): Ranking of genotypes in each environment by AMMI analysis**

| **Environment** | **Rank** | **Tree Height (m)** | | **Stem Girth(cm)** | | **Tree Spread(m)** | |
| --- | --- | --- | --- | --- | --- | --- | --- |
|  |  | **Genotype** | **Mean** | **Genotype** | **Mean** | **Genotype** | **Mean** |
| **Bhubaneshwar** | 1 | Vengurle-7 | 5.12 | Vengurle-7 | 76.25 | Vengurle-7 | 8.76 |
|  | 2 | Goa-1 | 4.86 | BPP-8 | 69.75 | BPP-8 | 7.42 |
|  | 3 | BPP-8 | 4.76 | Dhana | 68.91 | Priyanka | 7.08 |
|  | 4 | Bhaskara | 4.74 | Bhaskara | 64.26 | Dhana | 6.94 |
|  | 5 | Ullal-3 | 4.56 | Kanaka | 63.26 | Bhaskara | 6.18 |
|  | 6 | Madakkathara-1 | 4.53 | Madakkathara-1 | 60.57 | Vengurle-4 | 5.95 |
|  | 7 | Priyanka | 4.49 | Priyanka | 59.28 | Ullal-3 | 5.92 |
|  | 8 | Dhana | 4.47 | NRCC Sel-2 | 59.05 | NRCC Sel-2 | 5.90 |
|  | 9 | UN-50 | 4.46 | UN-50 | 57.74 | Kanaka | 5.84 |
|  | 10 | Vengurle-4 | 4.42 | Bhubaneswar-1 | 57.32 | Bhubaneswar-1 | 5.81 |
|  | 11 | Kanaka | 4.40 | Goa-1 | 57.03 | Madakkathara-1 | 5.78 |
|  | 12 | Ullal-4 | 4.37 | Vengurle-4 | 55.94 | Goa-1 | 5.69 |
|  | 13 | NRCC Sel-2 | 4.35 | Ullal-4 | 55.86 | Ullal-4 | 5.67 |
|  | 14 | K-22-1 | 4.28 | K-22-1 | 54.96 | UN-50 | 5.39 |
|  | 15 | Madakkathara-2 | 4.12 | Ullal-3 | 50.90 | K-22-1 | 5.39 |
|  | 16 | Bhubaneswar-1 | 3.85 | Madakkathara-2 | 50.42 | VRI-3 | 5.21 |
|  | 17 | Amrutha | 3.84 | VRI-3 | 47.92 | Madakkathara-2 | 4.77 |
|  | 18 | VRI-3 | 3.74 | Amrutha | 46.78 | Amrutha | 4.63 |
|  | **Mean** |  | **4.41** |  | **58.68** |  | **6.02** |
| **Jhargram** | 1 | Vengurle-7 | 6.09 | Dhana | 75.99 | Priyanka | 7.18 |
|  | 2 | BPP-8 | 5.63 | K-22-1 | 73.48 | Dhana | 6.98 |
|  | 3 | Goa-1 | 5.53 | Vengurle-7 | 70.07 | Vengurle-7 | 6.88 |
|  | 4 | Bhaskara | 5.24 | Goa-1 | 68.96 | Bhaskara | 6.87 |
|  | 5 | Dhana | 5.13 | NRCC Sel-2 | 68.07 | Vengurle-4 | 6.78 |
|  | 6 | Ullal-3 | 5.13 | Bhaskara | 65.43 | Goa-1 | 6.73 |
|  | 7 | Madakkathara-1 | 5.02 | UN-50 | 64.44 | BPP- | 6.67 |
|  | 8 | Priyanka | 4.97 | Madakkathara-2 | 63.10 | Ullal-4 | 6.58 |
|  | 9 | Vengurle-4 | 4.89 | Ullal-3 | 63.01 | Kanaka | 6.52 |
|  | 10 | UN-50 | 4.88 | Kanaka | 62.49 | VRI-3 | 6.39 |
|  | 11 | K-22-1 | 4.86 | Ullal-4 | 62.48 | Madakkathara-2 | 6.38 |
|  | 12 | Kanaka | 4.83 | Priyanka | 60.46 | Ullal-3 | 6.38 |
|  | 13 | NRCC Sel-2 | 4.68 | BPP-8(2/16) | 58.00 | K-22-1 | 6.36 |
|  | 14 | Ullal-4 | 4.58 | Madakkathara-1 | 56.04 | UN-50 | 6.31 |
|  | 15 | Madakkathara-2 | 4.57 | Vengurle-4 | 52.57 | NRCC Sel-2 | 6.07 |
|  | 16 | Amrutha | 4.02 | VRI-3 | 50.47 | Madakkathara-1 | 5.47 |
|  | 17 | Bhubaneswar-1 | 4.02 | Amrutha | 47.96 | Bhubaneswar-1 | 5.45 |
|  | 18 | VRI-3 | 3.98 | Bhubaneswar-1 | 42.97 | Amrutha | 5.44 |
|  | **Mean** |  | **4.89** |  | **61.44** |  | **6.41** |
| **Pilicode** | 1 | Madakkathara-2 | 7.04 | Kanaka | 75.70 | Ullal-4 | 7.60 |
|  | 2 | UN-50 | 7.01 | Madakkathara-1 | 74.50 | Bhubaneswar-1 | 7.11 |
|  | 3 | Vengurle-7 | 6.70 | UN-50 | 71.60 | Amrutha | 6.91 |
|  | 4 | Ullal-4 | 6.41 | Amrutha | 66.92 | Vengurle-7 | 6.87 |
|  | 5 | Amrutha | 5.92 | Ullal-4 | 66.00 | UN-50 | 6.56 |
|  | 6 | Bhubaneswar-1 | 5.55 | Madakkathara-2 | 65.42 | Kanaka | 6.30 |
|  | 7 | K-22-1 | 5.29 | Bhubaneswar-1 | 64.00 | K-22-1 | 6.28 |
|  | 8 | Goa-1 | 5.27 | Vengurla-7 | 61.00 | Ullal-3 | 6.20 |
|  | 9 | Ullal-3 | 5.25 | Goa-1 | 60.75 | BPP-8 | 6.04 |
|  | 10 | VRI-3 | 5.23 | K-22-1 | 53.50 | Madakkathara-1 | 6.01 |
|  | 11 | BPP-8 | 5.23 | Dhana | 53.25 | Goa-1 | 5.44 |
|  | 12 | Kanaka | 4.93 | BPP-8 | 53.25 | Madakkathara-2 | 5.30 |
|  | 13 | Madakkathara-1 | 4.90 | Bhaskara | 52.50 | NRCC Sel-2 | 5.08 |
|  | 14 | Bhaskara | 4.81 | Priyanka | 50.90 | Bhaskara | 5.00 |
|  | 15 | Dhana | 4.56 | NRCC Sel-2 | 48.50 | Dhana | 4.98 |
|  | 16 | NRCC Sel-2 | 4.25 | Ullal-3 | 46.50 | Priyanka | 4.94 |
|  | 17 | Priyanka | 4.20 | VRI-3 | 44.00 | VRI-3 | 4.93 |
|  | 18 | Vengurle-4 | 3.93 | Vengurla-4 | 33.33 | Vengurle-4 | 4.11 |
|  | **Mean** |  | **5.36** |  | **57.87** |  | **5.87** |
| **Vridhachallam** | 1 | Ullal-4 | 3.92 | Madakkathara-2 | 47.92 | Ullal-4 | 5.80 |
|  | 2 | Bhaskara | 3.66 | Madakkathara-1 | 47.57 | Kanaka | 5.27 |
|  | 3 | UN-50 | 3.61 | NRCC Sel-2 | 47.23 | UN-50 | 5.27 |
|  | 4 | NRCC Sel-2 | 3.59 | K-22-1 | 46.59 | Vengurle-7 | 5.24 |
|  | 5 | Goa-1 | 3.49 | Vengurle-7 | 46.55 | K-22-1 | 5.21 |
|  | 6 | Madakkathara-1 | 3.48 | Ullal-3 | 46.35 | Goa-1 | 5.17 |
|  | 7 | Kanaka | 3.46 | Vengurle-4 | 46.25 | Priyanka | 5.13 |
|  | 8 | Bhubaneswar-1 | 3.46 | Dhana | 46.17 | Ullal-3 | 5.10 |
|  | 9 | Priyanka | 3.44 | Goa-1 | 45.96 | Bhaskara | 5.05 |
|  | 10 | Amrutha | 3.42 | Ullal-4 | 45.94 | BPP-8 | 5.02 |
|  | 11 | Ullal-3 | 3.39 | Kanaka | 45.70 | Madakkathara-2 | 5.01 |
|  | 12 | Vengurle-4 | 3.39 | UN-50 | 45.44 | Dhana | 4.99 |
|  | 13 | Madakkathara-2 | 3.23 | VRI-3 | 45.17 | Amrutha | 4.80 |
|  | 14 | Vengurle-7 | 3.22 | Priyanka | 45.16 | VRI-3 | 4.80 |
|  | 15 | VRI-3 | 3.21 | Amrutha | 45.09 | Vengurle-4 | 4.71 |
|  | 16 | Dhana | 3.11 | BPP-8(2/16) | 44.99 | Bhubaneswar-1 | 4.67 |
|  | 17 | K-22-1 | 3.10 | Bhaskara | 44.75 | NRCC Sel-2 | 4.46 |
|  | 18 | BPP-8 | 3.01 | Bhubaneswar-1 | 44.40 | Madakkathara-1 | 4.30 |
|  | **Mean** |  | **3.40** |  | **45.96** |  | **5.00** |

**Table 4: (Supplementary) Contd: Ranking of genotypes in each environment by AMMI analysis**

| **Environment** | **Rank** | **Flowering Laterals/m^2^** | | **Sex Ratio** | | **Nuts/panicle** | |
| --- | --- | --- | --- | --- | --- | --- | --- |
|  |  | **Genotype** | **Mean** | **Genotype** | **Mean** | **Genotype** | **Mean** |
| **Bhubaneshwar** | 1 | VRI-3 | 23.29 | Kanaka | 0.90 | Bhubaneswar-1 | 6.59 |
|  | 2 | Dhana | 21.93 | Vengurle-4 | 0.55 | Vengurle-4 | 5.49 |
|  | 3 | Goa-1 | 21.58 | Bhaskara | 0.46 | Madakkathara-1 | 5.08 |
|  | 4 | Ullal-4 | 21.4 | Dhana | 0.35 | K-22-1 | 4.51 |
|  | 5 | Kanaka | 20.35 | Bhubaneswar-1 | 0.34 | NRCC Sel-2 | 4.13 |
|  | 6 | Vengurle-7 | 19.95 | VRI-3 | 0.30 | Ullal-3 | 4.07 |
|  | 7 | Priyanka | 19.31 | Madakkathara-1 | 0.25 | Ullal-4 | 4.05 |
|  | 8 | Bhaskara | 19.13 | Madakkathara-2 | 0.24 | VRI-3 | 4.03 |
|  | 9 | Vengurle-4 | 19.07 | Vengurle-7 | 0.21 | Goa-1 | 4.00 |
|  | 10 | BPP-8 | 18.75 | Ullal-3 | 0.19 | Amrutha | 3.91 |
|  | 11 | Madakkathara-1 | 18.63 | NRCC Sel-2 | 0.16 | Dhana | 3.81 |
|  | 12 | Amrutha | 18.54 | BPP-8 | 0.12 | Bhaskara | 3.63 |
|  | 13 | UN-50 | 18.1 | Goa-1 | 0.10 | UN-50 | 3.54 |
|  | 14 | NRCC Sel-2 | 17.95 | K-22-1 | 0.09 | BPP-8 | 3.43 |
|  | 15 | K-22-1 | 17.27 | Priyanka | 0.07 | Madakkathara-2 | 3.22 |
|  | 16 | Ullal-3 | 16.31 | Amrutha | 0.06 | Vengurle-7 | 2.70 |
|  | 17 | Bhubaneswar-1 | 15.74 | UN-50 | 0.06 | Kanaka | 2.13 |
|  | 18 | Madakkathara-2 | 15.14 | Ullal-4 | 0.04 | Priyanka | 0.68 |
|  | **Mean** |  | **19.02** |  | **0.25** |  | **3.83** |
| **Jhargram** | 1 | Madakkathara-2 | 26.36 | UN-50 | 0.65 | Bhubaneswar-1 | 11.43 |
|  | 2 | NRCC Sel-2 | 19.43 | Priyanka | 0.55 | Vengurle-4 | 8.76 |
|  | 3 | VRI-3 | 12.04 | Dhana | 0.47 | K-22-1 | 8.24 |
|  | 4 | Bhubaneswar-1 | 11.8 | Kanaka | 0.47 | NRCC Sel-2 | 8.03 |
|  | 5 | Bhaskara | 11.04 | Ullal-3 | 0.47 | Goa-1 | 8.01 |
|  | 6 | Vengurle-7 | 10.83 | NRCC Sel-2 | 0.44 | Ullal-3 | 7.83 |
|  | 7 | UN-50 | 10.48 | VRI-3 | 0.44 | Dhana | 7.77 |
|  | 8 | Ullal-3 | 9.89 | Ullal-4 | 0.41 | Amrutha | 7.06 |
|  | 9 | Amrutha | 9.7 | Bhaskara | 0.39 | BPP-8 | 6.80 |
|  | 10 | Goa-1 | 9.52 | Goa-1 | 0.35 | VRI-3 | 6.46 |
|  | 11 | BPP-8 | 8.51 | Madakkathara-1 | 0.31 | Bhaskara | 5.40 |
|  | 12 | Madakkathara-1 | 8.46 | Bhubaneswar-1 | 0.31 | UN-50 | 5.37 |
|  | 13 | Ullal-4 | 8.11 | Vengurle-4 | 0.28 | Madakkathara-2 | 5.21 |
|  | 14 | K-22-1 | 7.34 | Vengurle-7 | 0.25 | Vengurle-7 | 5.10 |
|  | 15 | Priyanka | 7.27 | BPP-8 | 0.21 | Ullal-4 | 5.09 |
|  | 16 | Kanaka | 7.01 | K-22-1 | 0.21 | Madakkathara-1 | 4.94 |
|  | 17 | Vengurle-4 | 6.94 | Amrutha | 0.15 | Kanaka | 4.40 |
|  | 18 | Dhana | 6.43 | Madakkathara-2 | 0.08 | Priyanka | 3.37 |
|  | **Mean** |  | **10.62** |  | **0.36** |  | **6.63** |
| **Pilicode** | 1 | Bhubaneswar-1 | 20.16 | Vengurle-7 | 0.17 | Madakkathara-1 | 13.04 |
|  | 2 | Ullal-4 | 17.77 | Priyanka | 0.16 | Ullal-4 | 9.90 |
|  | 3 | K-22-1 | 12.57 | VRI-3 | 0.16 | Bhaskara | 7.42 |
|  | 4 | Kanaka | 12.01 | BPP-8 | 0.13 | UN-50 | 6.61 |
|  | 5 | Priyanka | 11.70 | NRCC Sel-2 | 0.13 | Madakkathara-2 | 6.00 |
|  | 6 | Amrutha | 11.66 | Vengurle-4 | 0.13 | VRI-3 | 5.38 |
|  | 7 | Vengurle-7 | 11.25 | Bhubaneswar-1 | 0.12 | Vengurle-4 | 4.80 |
|  | 8 | UN-50 | 10.97 | Amrutha | 0.11 | Vengurle-7 | 4.54 |
|  | 9 | Madakkathara-1 | 10.68 | Goa-1 | 0.11 | Kanaka | 4.37 |
|  | 10 | Bhaskara | 9.54 | Madakkathara-1 | 0.11 | Amrutha | 3.75 |
|  | 11 | BPP-8 | 8.71 | Bhaskara | 0.11 | K-22-1 | 2.77 |
|  | 12 | Madakkathara-2 | 7.74 | Dhana | 0.10 | BPP-8 | 2.60 |
|  | 13 | NRCC Sel-2 | 6.42 | Madakkathara-2 | 0.09 | Ullal-3 | 2.19 |
|  | 14 | Goa-1 | 6.36 | Ullal-3 | 0.09 | Priyanka | 2.00 |
|  | 15 | Ullal-3 | 6.02 | UN-50 | 0.09 | NRCC Sel-2 | 1.88 |
|  | 16 | Dhana | 5.89 | Ullal-4 | 0.07 | Bhubaneswar-1 | 1.64 |
|  | 17 | Vengurle-4 | 5.21 | K-22-1 | 0.06 | Dhana | 1.63 |
|  | 18 | VRI-3 | 4.69 | Kanaka | 0.05 | Goa-1 | 1.46 |
|  | **Mean** |  | **9.96** |  | **0.11** |  | **4.55** |
| **Vridhachallam** | 1 | Madakkathara-2 | 19.3 | Vengurle-7 | 0.29 | Ullal-4 | 11.42 |
|  | 2 | NRCC Sel-2 | 17.9 | VRI-3 | 0.28 | Bhaskara | 9.47 |
|  | 3 | VRI-3 | 17.8 | Priyanka | 0.26 | Dhana | 6.49 |
|  | 4 | Goa-1 | 15.61 | Vengurle-4 | 0.26 | Amrutha | 6.03 |
|  | 5 | Vengurle-7 | 15.07 | BPP-8 | 0.26 | K-22-1 | 5.87 |
|  | 6 | Bhaskara | 14.74 | Bhubaneswar-1 | 0.25 | Vengurle-4 | 5.72 |
|  | 7 | Ullal-4 | 14.47 | Amrutha | 0.24 | Priyanka | 5.50 |
|  | 8 | Dhana | 14.45 | NRCC Sel-2 | 0.24 | Kanaka | 5.50 |
|  | 9 | UN-50 | 13.83 | Madakkathara-1 | 0.24 | Vengurle-7 | 5.31 |
|  | 10 | Amrutha | 13.71 | Bhaskara | 0.23 | Madakkathara-2 | 4.93 |
|  | 11 | Kanaka | 13.56 | Goa-1 | 0.23 | NRCC Sel-2 | 4.79 |
|  | 12 | BPP-8 | 13.41 | Madakkathara-2 | 0.22 | Bhubaneswar-1 | 4.63 |
|  | 13 | Madakkathara-1 | 13.25 | Dhana | 0.21 | Ullal-3 | 4.60 |
|  | 14 | Priyanka | 13.08 | Ullal-3 | 0.20 | Madakkathara-1 | 4.56 |
|  | 15 | Vengurle-4 | 13.02 | Kanaka | 0.18 | BPP-8 | 4.54 |
|  | 16 | Bhubaneswar-1 | 12.71 | K-22-1 | 0.18 | UN-50 | 4.45 |
|  | 17 | Ullal-3 | 12.69 | UN-50 | 0.18 | Goa-1 | 4.40 |
|  | 18 | K-22-1 | 11.88 | Ullal-4 | 0.18 | VRI-3 | 3.165 |
|  | **Mean** |  | **14.47** |  | **0.23** |  | **5.63** |

**Table 4: (Supplementary) Contd: Ranking of genotypes in each environment by AMMI analysis**

| **Environment** | **Rank** | **Nut Weight (g)** | | **Shelling Percentage** | | **Nut Yield (kg/tree)** | |
| --- | --- | --- | --- | --- | --- | --- | --- |
|  |  | **Genotype** | **Mean** | **Genotype** | **Mean** | **Genotype** | **Mean** |
| **Bhubaneshwar** | 1 | Vengurle-7 | 10.16 | VRI-3 | 33.81 | BPP-8 | 16.34 |
|  | 2 | Priyanka | 9.87 | Goa-1 | 33.32 | Vengurle-7 | 16.04 |
|  | 3 | Ullal-3 | 8.48 | Madakkathara-2 | 33.23 | Madakkathara-1 | 9.93 |
|  | 4 | UN-50 | 8.33 | Bhubaneswar-1 | 32.94 | Bhaskara | 8.80 |
|  | 5 | Dhana | 8.07 | K-22-1 | 32.07 | Dhana | 8.65 |
|  | 6 | NRCC Sel-2 | 7.86 | UN-50 | 31.94 | Bhubaneswar-1 | 8.08 |
|  | 7 | Ullal-4 | 7.71 | Vengurle-4 | 31.92 | Vengurle-4 | 7.75 |
|  | 8 | BPP-8 | 7.70 | Kanaka | 31.57 | Ullal-4 | 7.45 |
|  | 9 | Goa-1 | 7.70 | Vengurle-7 | 31.55 | K-22-1 | 6.20 |
|  | 10 | Madakkathara-2 | 7.39 | Madakkathara-1 | 31.21 | Kanaka | 5.95 |
|  | 11 | Amrutha | 7.30 | Ullal-3 | 31.16 | NRCC Sel-2 | 5.94 |
|  | 12 | Bhaskara | 7.08 | NRCC Sel-2 | 30.87 | Amrutha | 5.92 |
|  | 13 | Vengurle-4 | 6.85 | Amrutha | 30.41 | Ullal-3 | 4.94 |
|  | 14 | K-22-1 | 6.70 | Priyanka | 30.22 | Goa-1 | 4.78 |
|  | 15 | Madakkathara-1 | 6.53 | Bhaskara | 29.53 | VRI-3 | 4.34 |
|  | 16 | Kanaka | 6.25 | Ullal-4 | 29.48 | Madakkathara-2 | 4.04 |
|  | 17 | VRI-3 | 6.18 | BPP-8 | 29.37 | UN-50 | 3.30 |
|  | 18 | Bhubaneswar-1 | 6.18 | Dhana | 28.89 | Priyanka | 2.65 |
|  | **Mean** |  | **7.58** |  | **31.31** |  | **7.28** |
| **Jhargram** | 1 | Priyanka | 8.27 | VRI-3 | 35.83 | Vengurle-4 | 10.15 |
|  | 2 | Vengurle-7 | 8.06 | Bhubaneswar-1 | 35.17 | Bhaskara | 9.42 |
|  | 3 | UN-50 | 6.84 | Ullal-4 | 34.84 | Goa-1 | 9.35 |
|  | 4 | Ullal-3 | 6.82 | Madakkathara-1 | 34.49 | Vengurle-7 | 9.09 |
|  | 5 | Dhana | 6.67 | K-22-1 | 33.78 | Dhana | 8.69 |
|  | 6 | BPP-8 | 6.66 | Bhaskara | 33.67 | K-22-1 | 8.48 |
|  | 7 | NRCC Sel-2 | 6.48 | Goa-1 | 33.12 | BPP-8 | 8.46 |
|  | 8 | Amrutha | 6.41 | Vengurle-7 | 32.74 | NRCC Sel-2 | 8.23 |
|  | 9 | Ullal-4 | 6.32 | Ullal-3 | 32.66 | UN-50 | 7.62 |
|  | 10 | Madakkathara-2 | 6.19 | Madakkathara-2 | 32.14 | Ullal-3 | 7.59 |
|  | 11 | Goa-1 | 6.18 | Kanaka | 31.90 | Ullal-4 | 7.54 |
|  | 12 | Bhaskara | 5.91 | NRCC Sel-2 | 30.73 | VRI-3 | 7.27 |
|  | 13 | Vengurle-4 | 5.69 | Amrutha | 29.79 | Madakkathara-2 | 6.70 |
|  | 14 | VRI-3 | 5.61 | BPP-8 | 29.53 | Priyanka | 6.59 |
|  | 15 | K-22-1 | 5.56 | UN-50 | 29.49 | Bhubaneswar-1 | 5.75 |
|  | 16 | Kanaka | 5.36 | Vengurle-4 | 28.98 | Amrutha | 5.24 |
|  | 17 | Madakkathara-1 | 5.17 | Dhana | 27.33 | Kanaka | 4.97 |
|  | 18 | Bhubaneswar-1 | 5.10 | Priyanka | 26.74 | Madakkathara-1 | 4.14 |
|  | **Mean** |  | **6.29** |  | **31.83** |  | **7.52** |
| **Pilicode** | 1 | Priyanka | 10.88 | Madakkathara-1 | 34.28 | Kanaka | 9.47 |
|  | 2 | BPP-8 | 9.71 | Vengurle-7 | 33.34 | Bhubaneswar-1 | 8.17 |
|  | 3 | Amrutha | 9.63 | NRCC Sel-2 | 33.06 | Priyanka | 6.94 |
|  | 4 | VRI-3 | 9.01 | Ullal-4 | 32.73 | Madakkathara-2 | 6.36 |
|  | 5 | Vengurle-7 | 8.37 | Bhaskara | 32.27 | Ullal-4 | 5.78 |
|  | 6 | Dhana | 8.05 | Vengurle-4 | 31.65 | Bhaskara | 4.06 |
|  | 7 | NRCC Sel-2 | 7.88 | Dhana | 31.06 | BPP-8 | 3.59 |
|  | 8 | Kanaka | 7.83 | Amrutha | 30.97 | Ullal-3 | 2.81 |
|  | 9 | UN-50 | 7.77 | Priyanka | 30.85 | Goa-1 | 2.74 |
|  | 10 | Ullal-3 | 7.14 | BPP-8 | 30.10 | Madakkathara-1 | 2.55 |
|  | 11 | Madakkathara-2 | 7.13 | Ullal-3 | 29.99 | Amrutha | 2.51 |
|  | 12 | Ullal-4 | 6.97 | Kanaka | 29.53 | Vengurle-4 | 2.21 |
|  | 13 | Bhaskara | 6.81 | Goa-1 | 29.36 | VRI-3 | 1.78 |
|  | 14 | K-22-1 | 6.67 | K-22-1 | 28.95 | UN-50 | 1.77 |
|  | 15 | Goa-1 | 6.29 | Madakkathara-2 | 28.85 | NRCC Sel-2 | 1.08 |
|  | 16 | Vengurle-4 | 6.07 | Bhubaneswar-1 | 28.38 | K-22-1 | 1.06 |
|  | 17 | Bhubaneswar-1 | 5.92 | UN-50 | 28.26 | Dhana | 0.78 |
|  | 18 | Madakkathara-1 | 5.13 | VRI-3 | 28.01 | Vengurle-7 | 0.71 |
|  | **Mean** |  | **7.62** |  | **30.65** |  | **3.58** |
| **Vridhachallam** | 1 | Priyanka | 7.41 | Vengurle-4 | 33.49 | Vengurle-4 | 7.48 |
|  | 2 | Amrutha | 6.99 | Vengurle-7 | 32.82 | Bhaskara | 7.46 |
|  | 3 | Madakkathara-2 | 6.99 | NRCC Sel-2 | 32.74 | Goa-1 | 7.02 |
|  | 4 | Vengurle-7 | 6.95 | Madakkathara-1 | 32.43 | BPP-8 | 6.73 |
|  | 5 | BPP-8 | 6.95 | Priyanka | 32.39 | Ullal-4 | 6.56 |
|  | 6 | UN-50 | 6.94 | Goa-1 | 32.24 | Vengurle-7 | 6.41 |
|  | 7 | VRI-3 | 6.90 | Madakkathara-2 | 32.23 | Priyanka | 6.12 |
|  | 8 | Vengurle-4 | 6.83 | UN-50 | 31.71 | Madakkathara-2 | 6.06 |
|  | 9 | Dhana | 6.81 | Amrutha | 31.65 | Dhana | 6.06 |
|  | 10 | Bhaskara | 6.80 | Kanaka | 31.26 | K-22-1 | 5.95 |
|  | 11 | Ullal-3 | 6.77 | Dhana | 31.20 | Bhubaneswar-1 | 5.91 |
|  | 12 | Ullal-4 | 6.77 | VRI-3 | 31.15 | NRCC Sel-2 | 5.77 |
|  | 13 | NRCC Sel-2 | 6.64 | Ullal-3 | 30.90 | Ullal-3 | 5.77 |
|  | 14 | Goa-1 | 6.57 | Bhubaneswar-1 | 30.81 | Kanaka | 5.67 |
|  | 15 | K-22-1 | 6.42 | K-22-1 | 30.79 | UN-50 | 5.49 |
|  | 16 | Kanaka | 6.27 | BPP-8 | 30.44 | VRI-3 | 5.25 |
|  | 17 | Bhubaneswar-1 | 6.26 | Bhaskara | 30.32 | Amrutha | 4.00 |
|  | 18 | Madakkathara-1 | 6.00 | Ullal-4 | 30.13 | Madakkathara-1 | 3.27 |
|  | **Mean** |  | **6.74** |  | **31.59** |  | **5.94** |
